# Beamforming Design for STAR-RIS-Assisted NOMA with Binary and Coupled Phase-Shifts

**DOI:** 10.3390/e27020210

**Published:** 2025-02-17

**Authors:** Yongfei Liu, Yuhuan Wang, Weizhang Xu

**Affiliations:** 1 Engineering Research Center of Digital Audio and Video Ministry of Education, Communication University of China, Beijing 100024, China; lyf2020@cuc.edu.cn (Y.L.); wangyuhuan@cuc.edu.cn (Y.W.); 2 State Key Laboratory of Media Convergence and Communication, Communication University of China, Beijing 100024, China

**Keywords:** STAR-RIS NOMA, active beamforming, passive beamforming, FP, Nesterov’s extrapolation, binary phase design, system throughput optimization, convex optimization, linear time complexity

## Abstract

This paper investigates the joint optimization of active and passive beamforming in simultaneously transmitting and reflecting reconfigurable intelligent surface (STAR-RIS)-assisted non-orthogonal multiple access (NOMA) systems, with the aim of maximizing system throughput and improving overall performance. To achieve this goal, we propose an iterative and efficient algorithmic framework. For active beamforming optimization, the fractional programming (FP) method is employed to reformulate the non-convex optimization problem into a convex problem, making it more tractable. Additionally, Nesterov’s extrapolation technique is introduced to enhance the convergence rate and reduce computational overhead. For the phase optimization of the STAR-RIS, a binary phase design method is proposed, which reformulates the binary phase optimization problem as a segmentation problem on the unit circle. This approach enables a closed form solution that can be derived in linear time. Simulation results demonstrate that the proposed algorithmic framework outperforms existing benchmark algorithms in terms of both system throughput and computational efficiency, verifying its effectiveness and practicality in STAR-RIS-assisted NOMA systems.

## 1. Introduction

Driven by next-generation applications and the exponential growth of data traffic, the sixth generation (6G) wireless communication system faces unprecedented challenges. With constrained spectrum resources, achieving both high throughput and high reliability has become a central focus for academia and industry. In this context, reconfigurable intelligent surfaces (RISs) have garnered significant attention due to their near-passive operation, energy efficiency, and ease of deployment, emerging as a promising solution to enhance spectrum utilization and expand network coverage [1,2].

However, despite these advantages, traditional RISs have a significant limitation: their operation requires the transmitter and receiver to be located on the same side of the RIS. In practical scenarios, this layout is not always feasible. For example, when the transmitter and receiver are positioned on opposite sides of the surface, the signal may need to rely on multiple RISs as relays, undergoing multiple reflections before reaching the target. This multi-reflection process not only results in significant propagation losses but also severely degrades the overall system performance and reliability.

To overcome this, simultaneously transmitting and reflecting RIS (STAR-RISs) have been introduced. Unlike conventional RISs, each element of STAR-RISs can independently control both transmitted and reflected signals, enabling 360-degree full-space coverage. This unique feature overcomes the geographical constraints of a traditional RIS, making it more flexible for diverse application scenarios [3]. Additionally, STAR-RISs introduce a higher degrees of freedom (DoF) by simultaneously adjusting the transmission and reflection coefficients, further optimizing the performance and adaptability of wireless networks.

Meanwhile, non-orthogonal multiple access (NOMA) technology has garnered increasing attention for its ability to support multiple users within the same time-frequency resource, significantly improving spectrum efficiency [4]. However, the performance of NOMA largely depends on the channel condition disparities among users. This is where a traditional reflective RIS falls short, as its reflection-only property often leads to similar channel conditions for users, thereby limiting the performance gains of NOMA. In contrast, integrating NOMA with STAR-RISs leverages its dual transmission and reflection capabilities to dynamically tailor channel conditions for users in different directions. This unique flexibility enables STAR-RISs to optimize signal propagation paths, meeting NOMA’s requirement for channel condition disparities and thereby, fully unlocking its spectrum efficiency potential.

Nevertheless, the integration of STAR-RISs and NOMA introduces intricate optimization challenges. The design of transmission and reflection coefficients at STAR-RISs and active precoding at base stations forms a coupled non-convex problem. Furthermore, the phase vector constraints of STAR-RISs, along with the maximum power constraints, further increase the complexity of this non-convex optimization problem. Existing optimization frameworks often struggle to balance computational feasibility and performance optimality, especially in large-scale deployments. Therefore, researching efficient joint resource allocation algorithms to coordinate the resource allocation of STAR-RIS NOMA remains an optimization problem that needs to be urgently addressed.

### 1.1. Related Works

Recent advancements in STAR-RIS-based communication systems have shown notable progress in optimizing resource allocation. In STAR-RIS-aided communication systems, efficiently designing resource allocation strategies to maximize system performance is a key direction for research. Existing works primarily adopt alternating optimization frameworks to address the coupled challenges of active and passive beamforming design. For instance, Ref. [5] proposed an optimization algorithm that alternates between active and passive beamforming. For ideal intelligent reflecting surfaces (IRSs) scenarios, continuous convex approximation techniques were employed to solve the two subproblems. For non-ideal IRS scenarios, IRS elements with a constant modulus were further divided into continuous and discrete phase shifts. Building on this, Ref. [6] presented an optimization framework for joint channel allocation, transmission and reflection coefficients, power allocation, and decoding order in STAR-RIS NOMA systems, formulating a mixed-integer nonlinear programming (MINLP) model. Their framework employs convex upper bound (CUB) methods for transmission and reflection coefficient optimization, along with geometric programming (GP) for power allocation, achieving near-optimal solutions under continuous phase assumptions. In Ref. [7], a STAR-RIS-assisted NOMA system was investigated, considering the case of asymmetric transmission and reflection users. Four phase configuration strategies were proposed, while Ref. [8] studied STAR-RIS-assisted MISO systems and proposed a coupled phase-shift model. The authors employed hybrid deep deterministic policy gradient (DDPG) and joint DDPG–deep Q-network (DDPG–DQN) reinforcement learning algorithms to optimize transmission power consumption. Notably, Ref. [9] jointly optimized ISAC base station beamforming and STAR-RIS passive beamforming to maximize the weighted sum rate of communication users. To address this non-convex optimization problem, an alternating optimization approach was adopted. The base station beamforming problem was reformulated into a weighted minimum mean square error (WMMSE) problem, while the STAR-RIS beamforming problem was equivalently transformed into an FP problem. These subproblems were further converted into solvable semidefinite programming (SDP) problems using semidefinite relaxation (SDR) techniques. Ref. [10] investigated the energy efficiency (EE) optimization of MISO STAR-RIS-aided NOMA downlink networks. By alternating the optimization of phase shifts and beamforming, phase shift optimization employed FP to transform the sum rate maximization problem into an SDP problem with rank-1 constraints. Additionally, a novel sequential rank-1constraint relaxation (SROCR) method was proposed, which effectively converted rank-1 constraints into convex constraints, overcoming the limitations of Gaussian randomization. Beamforming optimization was also solved using FP and SDR techniques. In parallel, Ref. [11] focused on mobile STAR-RIS-aided NOMA systems, proposing a joint optimization of phase shifts, channel allocation, and STAR-RIS positioning to maximize the uplink average sum rate. This scheme used SCA methods for position optimization and SDP methods for phase shift optimization. Ref. [12] explored the potential of UAV-assisted wireless networks combined with STAR-RISs, leveraging the transmission and reflection characteristics of STAR-RISs for full-space coverage. For STAR-RIS-assisted NOMA UAV networks, a sum rate maximization problem was formulated. UAV positioning was optimized using SCA, transmission beamforming was analytically solved, and reflection beamforming was solved using CVX tools after SDR conversion.

While the above studies have made significant progress in optimizing STAR-RIS systems, most focus on the assumption of continuous phase shifts. However, existing research indicates that when the quantization resolution exceeds 3 bits, performance loss due to quantization is typically negligible, as shown in Ref. [6]. In practical hardware designs, due to complexity and resource constraints, phase shifts often need to be quantized into discrete values. In low-resolution scenarios (e.g., 1-bit quantization with only two-phase states), traditional optimization methods exhibit the following limitations:

- High computational complexity: While an exhaustive search can achieve globally optimal solutions, they suffer from exponential complexity growth, making them impractical for large-scale scenarios.
- Performance degradation: Despite their computational efficiency, relaxation-based discretization techniques lack theoretical performance guarantees and might suffer from severe performance loss in low-resolution situations.

Therefore, developing optimization algorithms for 1-bit discrete phase shifts is of great significance.

### 1.2. Contributions

To address these pressing challenges, this paper makes the following key contributions:

- Development of an iterative optimization framework: This paper proposes an optimization framework to handle the coupling between variables in STAR-RIS-assisted NOMA systems, effectively balancing convergence speed and solution accuracy.
- Integration of FP and Nesterov’s extrapolation: During the active beamforming stage, we employ the FP algorithm to transform the original problem into a convex optimization problem, while simultaneously leveraging Nesterov’s extrapolation technique to reduce computational complexity. This approach ensures that the entire process maintains the convexity of the problem, while achieving efficient and stable beamforming optimization.
- Proposal of a binary phase design method: For the phase vector optimization problem of STAR-RISs, a binary phase design method with linear time complexity is proposed. This method reduces computational complexity and enhances feasibility by equivalently transforming the binary phase beamforming problem into a piecewise solution problem on the unit circle and deriving an optimal closed-form solution.

### 1.3. Organization

The structure of this paper is organized as follows: Section 2 details the system model and problem formulation. Section 3 introduces the proposed iterative optimization algorithm and its core techniques. Section 4 presents the simulation results. Section 5 concludes the paper, discusses the limitations of the study, and outlines future research directions.

## 2. System Model and Problem Formulation

### 2.1. System Model

**Figure 1:**
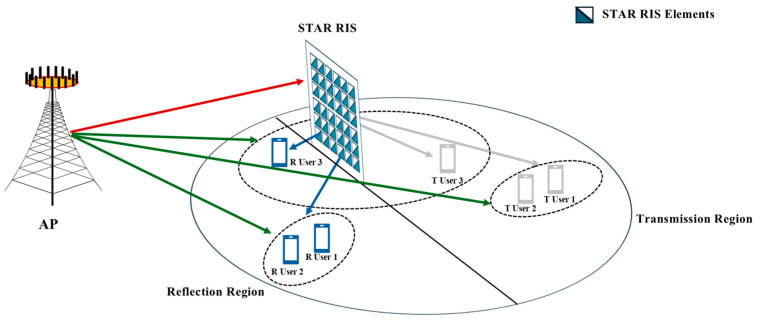
System model of a STAR-RIS-assisted NOMA network.

The system under consideration is a STAR-RIS-assisted NOMA communication network comprising the following key components: as shown in Figure 1, the communication system is equipped with an access point (AP) that has K independent antennas, enabling parallel transmission across K subchannels within the same time slot. In systems supported by NOMA, each subchannel can simultaneously serve multiple users. In this study, we assume that each subchannel can concurrently support two users. Consequently, I=2K single-antenna users are evenly allocated to the K subchannels, accommodating a total of 2K users.

The STAR-RIS consists of M individually controllable elements. Each unit of the STAR-RIS can control the signal in both transmission and reflection directions. Specifically, each element has a transmission coefficient $\beta_{m}^{t}\in \left[0,1\right],m\in M$ and a reflection coefficient $\beta_{m}^{r}\in \left[0,1\right],m\in M$, where $M=\left\{1,\cdots ,M\right\}$. These coefficients represent the amplitude adjustments applied by the m-th element to the incident signal during transmission and reflection. According to the principle of energy conservation, the sum of the energies of the transmitted and reflected signals equals the energy of the incident signal exerted on each element. This relationship is given by ${\left(\beta_{m}^{t}\right)}^{2}+{\left(\beta_{m}^{r}\right)}^{2}=1$, as provided in Ref. [3]. In practical applications, the amplitudes and phases of the STAR-RIS elements depend on their electrical and magnetic impedance characteristics. Based on the analysis in Ref. [13], for a passive and lossless STAR-RIS, the following condition must be satisfied: $\cos\left(\varphi_{m}^{t}-\varphi_{m}^{r}\right)=0$, $\varphi_{m}^{t},\varphi_{m}^{r}\in \left[0,2\pi \right)$, m∈M. This condition ensures energy conservation and enforces a 90-degree or 270-degree phase difference between transmission and reflection. By jointly optimizing these amplitudes and phases, the STAR-RIS can achieve accurate regulation of the amplitude and direction of the received signals in both the transmission and reflection domains.

Furthermore, $v_{n}={\left[\beta_{1}^{n}\theta_{1}^{n},\beta_{2}^{n}\theta_{2}^{n},\cdots ,\beta_{M}^{n}\theta_{M}^{n}\right]}^{T}$, $\theta_{m}^{n}=e^{j\varphi_{m}^{n}},n\in \left\{t,r\right\}$ represent the transmission and reflection vectors of the STAR-RIS, with amplitude coefficients $\beta_{m}^{n}$ and phase adjustments $\theta_{m}^{n}$. Additionally, let $g_{k}\in {\mathbb{C}}^{M\times K}$, and $f_{k,i}\in {\mathbb{C}}^{M\times 1}$ denote the narrowband quasi-static Rician fading channels from the AP to the STAR-RIS and from the STAR-RIS to the users, respectively. And $h_{k,i}^{d}$ denotes the direct channel vector received by the i-th user from the k-th AP subchannel. In this system, the AP optimizes the signal transmission paths for all users by designing a unified precoding matrix W. Specifically, the precoding vectors for all users within each group are identical, and these vectors collectively form the precoding matrix for each subchannel.

Based on their locations, users are divided into transmission-region users (T users) and reflection-region users (R users), forming the sets $T=\left\{T_{1},\cdots ,T_{K}\right\}$ and $R=\left\{R_{1},\cdots ,R_{K}\right\}$, respectively. For simplicity, it is assumed that the number of users in the transmission and reflection regions is equal. This assumption simplifies the analysis but does not limit the generality of the proposed algorithm, which can readily accommodate scenarios with asymmetric user distributions between the two regions.

### 2.2. Mathematical Model of the System

The channel for user i on subchannel k can be expressed as follows:

(1) $$h_{k,i}=h_{k,i}^{d}+g_{k}^{H}diag(f_{k,i})v_{n}$$

This formulation captures the combined contributions of the direct channel $h_{k,i}^{d}$ and the STAR-RIS-assisted reflected channel, as represented by $g_{k}^{H}diag(f_{k,i})v_{n}$.

In the context of data transmission, the transmitted signal on subchannel k is defined as follows:

(2) $$x_{k}=\sum_{i=1}^{2}\sqrt{p_{k,i}}s_{k,i},$$

where $s_{k,i}$ denotes the symbol transmitted by the user i, normalized to satisfy $E\left[{\left|s_{k,i}\right|}^{2}\right]=1$, and $p_{k,i}$ represents the power allocated to this user. Upon reception, the received signal for user i on subchannel k can be expressed as follows:

(3) $$y_{k,i}=h_{k,i}^{H}w_{k}x_{k}+z_{k,i},$$

Here, $h_{k,i}$ is the effective channel gain, $w_{k}$ is the precoding vector for subchannel k, and $z_{k,i}$ denotes the additive white Gaussian noise (AWGN) with zero mean and variance $\sigma_{k}^{2}$.

To simplify the analysis, this paper does not explicitly consider inter-beam interference. Building on this, the system sum-rate can be derived as follows:

(4) $$R_{sum}=\sum_{k=1}^{K}\sum_{i=1}^{2}\log_{2}\left(1+\frac{{\left|h_{k,i}^{H}w_{k}\right|}^{2}p_{k.i}}{\sum_{l=1}^{i-1}p_{k.l}{\left|h_{k,i}^{H}w_{k}\right|}^{2}+\sigma_{k}^{2}}\right)$$

For clarity, this expression can be further expanded to the following:

(5) Rsum=∑k=1K∑i=12log21+hk,idHwk2+2Rehk,idHwkvnHqk,iHwk+vnHqk,iHwk2pk.i∑l=1i−1pk.lhk,idHwk2+2Rehk,idHwkvnHqk,iHwk+vnHqk,iHwk2+σk2

To fully exploit the potential of STAR-RIS NOMA systems, it is essential to jointly optimize the precoding vectors and the transmission and reflection vectors. This joint optimization must adhere to several physical constraints while maximizing the system sum-rate, leading to the following optimization problem:

(6a) $$\underset{\left\{w,v_{n}\right\}}{\max}R_{sum}$$

(6b) $$s.t.{\left(\beta_{m}^{t}\right)}^{2}+{\left(\beta_{m}^{r}\right)}^{2}=1,\forall m\in M,$$

(6c) $$\varphi_{m}^{n}\in \left\{0,\pi \right\},\forall m\in M,n\in \left\{t,r\right\},$$

(6d) $$\cos\left(\varphi_{m}^{t}-\varphi_{m}^{r}\right)=0,\forall m\in M,$$

(6e) $$\sum_{k=1}^{K}\sum_{i=1}^{2}p_{k,i}{\left\|w_{k}\right\|}^{2}\leq P_{\max},$$

Here, $w_{i}$ represents the precoding vector. Constraint Equation (6b) reflects the law of energy conservation, and defines a set of points on a unit circle, which is non-convex. Constraint Equations (6c) and (6d) jointly impose a binary phase condition on the transmission and reflection phase shifts of the STAR-RIS NOMA elements. Specifically, Equation (6c) restricts the transmission phase shift $\varphi_{m}^{n}$ to binary values $\left\{0,\pi \right\}$, while Equation (6d) ensures that the transmission and reflection phase shifts are orthogonal, such that $\varphi_{m}^{t}-\varphi_{m}^{r}=\pm \pi /2$. Together, these constraints guarantee that the STAR-RIS elements operate under a discrete and orthogonal phase design. Additionally, Constraint Equation (6e) imposes a total transmission power limit, ensuring that the maximum transmission power does not exceed $P_{\max}$.

By jointly optimizing the beamforming vector w and the STAR-RIS phase shift vector $v_{n}$, the proposed framework can effectively maximize the system sum-rate, while adhering to the physical and practical constraints of the STAR-RIS NOMA system. The optimization problem presents several key challenges:

1. Non-convex objective function: The system sum-rate is a non-convex function due to the coupling between beamforming and STAR-RIS phase shifts, making it difficult to find the global optimum.
2. Nonlinear constraints: Discrete phase shifts—practical hardware imposes discrete phase shifts, turning the problem into a mixed-integer optimization, which significantly increases computational complexity. Coupled phase shifts—passive STAR-RIS elements require orthogonal transmission and reflection phase shifts, $\cos(\varphi_{m}^{t}-\varphi_{m}^{r})=0$, restricting the phase difference to π/2 or 3π/2, introducing additional non-convexity and nonlinear equality constraints. The transmission and reflection coefficient constraints are non-convex because they define a unit circle, which is not a convex set.

These challenges highlight the inherent complexity of jointly optimizing beamforming and STAR-RIS NOMA configurations. In the following section, we present two algorithms aimed at solving these problems.

## 3. Proposed Optimization Frameworks

To address this non-convex optimization problem, we propose an optimization framework based on the alternating optimization of active and passive beamforming for STAR-RIS NOMA. Specifically, for active beamforming, we employ the FP algorithm with Nesterov’s acceleration to convert the non-convex problem into a convex optimization problem, reducing complexity. For passive beamforming, we derive a solution with linear complexity. The following two subsections will provide a detailed introduction to this optimization algorithm.

### 3.1. Active Beamforming Optimization for STAR-RIS NOMA

To address the non-convexity of the objective function in the active beamforming optimization problem, a dual transformation is applied. By introducing auxiliary variables $\alpha \in {\mathbb{R}}^{K\times 2}$, the optimization problem can be reformulated as follows [14]:

(7a) $$\underset{\left\{w,\alpha \right\}}{\max}\sum_{k=1}^{K}\sum_{i=1}^{2}\left[\log_{2}\left(1+\alpha_{k,i}\right)-\alpha_{k,i}+\left(1+\alpha_{k,i}\right)\frac{p_{k,i}{\left|h_{k,i}^{H}w_{k}\right|}^{2}}{\sum_{l=1}^{i}p_{k.l}{\left|h_{k,i}^{H}w_{k}\right|}^{2}+\sigma_{k}^{2}}\right]$$

(7b)  s.t. (6e)

where $\left\{\alpha_{k,i}\right\}$ is an auxiliary variable to facilitate optimization. The optimal solution of the objective function can be obtained by setting the derivative with respect to $\alpha_{k,i}$ to zero, as follows:

(8) $$\alpha_{k,i}^{o}=\frac{p_{k.i}{\left|h_{k,i}^{H}w_{k}\right|}^{2}}{\sum_{l=1}^{i-1}p_{k.l}{\left|h_{k,i}^{H}w_{k}\right|}^{2}+\sigma_{k}^{2}}$$

Substituting $\alpha_{k,i}^{o}$ into the objective function simplifies the expression to the following:

(9) $$f_{LD}\left(w\right)=\overset{K}{\underset{k=1}{\sum }}\overset{2}{\underset{i=1}{\sum }}\left[const\left(\alpha \right)+\left(1+\alpha_{k,i}^{o}\right)\frac{p_{k,i}{\left|h_{k,i}^{H}w_{k}\right|}^{2}}{\sum_{l=1}^{i}p_{k.l}{\left|h_{k,i}^{H}w_{k}\right|}^{2}+\sigma_{k}^{2}}\right]$$

Here, $const\left(\alpha \right)=\log_{2}\left(1+\alpha_{k,i}^{o}\right)-\alpha_{k,i}^{o}$ is a constant term. Since the last term in Equation (9) contains a fractional form, the multidimensional quadratic transformation is applied to further derive the following expression [14]:

(10) $$f_{MQ}\left(w,\chi \right)=\overset{K}{\underset{k=1}{\sum }}\overset{2}{\underset{i=1}{\sum }}\left[const\left(\alpha \right)+2\sqrt{\left(1+\alpha_{k,i}^{o}\right)p_{k,i}}Re\left\{\chi_{k,i}^{H}h_{k,i}^{H}w_{k}\right\}-{\left|\chi_{k,i}\right|}^{2}\left(\sum_{l=1}^{i}p_{k.l}{\left|h_{k,i}^{H}w_{k}\right|}^{2}+\sigma_{k}^{2}\right)\right].$$

where $\chi \in {\mathbb{C}}^{K\times 2}$ represents the set $\left\{\chi_{k,i}\right\}$. Similarly, following the same method as for $\left\{\alpha_{k,i}\right\}$, the optimal solution for $\left\{\chi_{k,i}\right\}$ can be expressed as follows:

(11) $$\chi_{k,i}^{o}=\frac{\sqrt{\left(1+\alpha_{k,i}\right)p_{k,i}}h_{k,i}^{H}w_{k}}{\sum_{l=1}^{i}p_{k.l}{\left|h_{k,i}^{H}w_{k}\right|}^{2}+\sigma_{k}^{2}}$$

By substituting (11) into (10), the resulting expression depends solely on $\left\{w_{m}\right\}$, as follows:

(12) $$f_{MQ}\left(w\right)=\overset{K}{\underset{k=1}{\sum }}\overset{2}{\underset{i=1}{\sum }}\left[const\left(\alpha \right)+2\sqrt{\left(1+\alpha_{k,i}^{o}\right)p_{k,i}}Re\left\{{\left(\chi_{k,i}^{o}\right)}^{H}h_{k,i}^{H}w_{k}\right\}-{\left|\chi_{k,i}^{o}\right|}^{2}\left(\sum_{l=1}^{i}p_{k.l}{\left|h_{k,i}^{H}w_{k}\right|}^{2}+\sigma_{k}^{2}\right)\right]$$

At this stage, the optimization problem with respect to $\left\{w_{m}\right\}$, subject to Constraint Equation (6e), can be equivalently transformed into an unconstrained optimization problem, as follows [15]:

(13) $$f_{FP}\left(w\right)=\overset{K}{\underset{k=1}{\sum }}\overset{2}{\underset{i=1}{\sum }}\left[2\sqrt{\left(1+\alpha_{k,i}^{o}\right)p_{k,i}}Re\left\{{\left(\chi_{k,i}^{o}\right)}^{H}h_{k,i}^{H}w_{k}\right\}-{\left|\chi_{k,i}^{o}\right|}^{2}\sum_{l=1}^{i}p_{k.l}{\left|h_{k,i}^{H}w_{k}\right|}^{2}-{\left|\chi_{k,i}^{o}\right|}^{2}\sigma_{k}^{2}\frac{\sum_{x=1}^{K}\sum_{y=1}^{2}p_{x,y}{\left\|w_{x}\right\|}^{2}}{P_{\max}}\right]$$

At this point, the original non-convex optimization problem has been reformulated into an unconstrained convex optimization problem, allowing the closed-form solution for $w_{m}^{o}$ to be derived as follows:

(14) $$w_{k}^{o}={\left(\overset{2}{\underset{i=1}{\sum }}{\left|\chi_{k,i}^{o}\right|}^{2}\sum_{l=1}^{i}p_{k.l}h_{k,i}h_{k,i}^{H}+\frac{\sum_{y=1}^{2}p_{k,y}\;}{P_{\max}}\overset{K}{\underset{k=1}{\sum }}\overset{2}{\underset{i=1}{\sum }}{\left|\chi_{k,i}^{o}\right|}^{2}\sigma_{k}^{2}I_{K}\right)}^{-1}\left(\overset{2}{\underset{i=1}{\sum }}\sqrt{\left(1+\alpha_{k,i}^{o}\right)p_{k,i}}h_{k‚i}\chi_{k,i}^{o}\right)$$

From the mathematical expression of this solution, it is evident that when the number of transmit antennas K is large, solving the problem involves repeatedly computing the inverse of high-dimensional matrices. To address this challenge, we leverage an approximation approach based on the methods of Ref. [16]. Specifically, by defining auxiliary variable t and the matrices L and $M\in H^{n},M≻=L$, the following equation holds:

(15a) −xHLx≥−xHMx−2RexHL−Mt+tHM−Lt

(15b) maxx∈ζ−xHLx⇔maxx∈ζ,t∈ζ−xHMx−2RexHL−Mt+tHM−Lt

where t representing the latest updated value of x.

To further reduce complexity, we define the following:

(16) $$D_{k}=\overset{2}{\underset{i=1}{\sum }}{\left|\chi_{k,i}^{o}\right|}^{2}\sum_{l=1}^{i}p_{k.l}h_{k,i}h_{k,i}^{H}+\overset{2}{\underset{i=1}{\sum }}{\left|\chi_{k,i}^{o}\right|}^{2}\frac{\sigma_{k}^{2}}{P_{\max}}\sum_{j=1}^{2}p_{k,j}I_{K},\;\mathrm{where}\;K_{k}=\lambda_{k}I_{K},\lambda_{k}\geq \lambda_{\max}\left(D_{k}\right)$$

subject to the condition $D_{k}≺=K_{k}$. Here, $\lambda_{\max}\left(D_{k}\right)$ represents the maximum eigenvalue of the matrix $D_{k}$, and is typically assigned as $\lambda_{k}=\lambda_{\max}\left(D_{k}\right)$. For each k, by applying Equation (15a) up to the second-order term $w_{k}^{H}D_{k}w_{k}$, the following expression can be derived:

(17) −wkHDkwk≥−wkHKkwk−2RewkHDk−Kktk−tkHKk−Dktk

Substituting Equation (17) into the objective function in Equation (13) yields the following inequality:

(18) $$\overset{K}{\underset{k=1}{\sum }}\left[-w_{k}^{H}D_{k}w_{k}+2\overset{2}{\underset{i=1}{\sum }}\sqrt{\left(1+\alpha_{k,i}^{o}\right)p_{k,i}}Re\left\{{\left(\chi_{k,i}^{o}\right)}^{H}h_{k,i}^{H}w_{k}\right\}-\overset{2}{\underset{i=1}{\sum }}{\left|\chi_{k,i}^{o}\right|}^{2}\frac{\sigma_{k}^{2}}{P_{\max}}\sum_{l\neq k}^{K}\sum_{j=1}^{2}p_{l,j}{\left\|w_{l}\right\|}^{2}\right]\geq f_{FP}(w,t)$$

With

(19) fFP(w,t)=∑k=1K−wkHKkwk−2RewkHDk−Kktk−tkHKk−Dktk−∑k=1K∑i=12χk,io2σk2Pmax∑l≠kK∑j=12pl,jwl2−21+αk,iopk,iReχk,ioHhk,iHwk.

The function $f_{FP}(w,t)$ is identified as a lower bound for the objective function $f_{FP}\left(w\right)$. Maximizing the lower bound function Equation (19) with respect to $w_{k}$ serves as an effective approximation for maximizing Equation (13). By taking the derivative of $w_{k}$ with respect to $f_{FP}(w,t)$ and setting it to zero, the optimal $w_{k}$ is derived in closed form, ensuring both efficiency and accuracy.

(20) $$w_{k}={\left[\left(\overset{2}{\underset{i=1}{\sum }}\sqrt{\left(1+\alpha_{k,i}^{o}\right)p_{k,i}}{\left(\chi_{k,i}^{o}\right)}^{H}h_{k,i}^{H}-t_{k}^{H}\left(D_{k}^{H}-{K_{k}}^{H}\right)\right)K_{k}^{-1}\right]}^{H}$$

In each iteration, the auxiliary variable $t_{k}$ is initialized using the $w_{k}$ value from the previous iteration, i.e., $t_{k}={w_{k}}^{\left(t\right)}$. Given that $K_{k}=\lambda_{k}I$, the inverse can be expressed as ${K_{k}}^{-1}=\frac{1}{\lambda_{k}}I$. Consequently, the update rule for $w_{k}$ is derived as follows:

(21) $${w_{k}}^{\left(t+1\right)}={\left(\frac{1}{\lambda_{k}}I\right)}^{H}\left(\overset{2}{\underset{i=1}{\sum }}\sqrt{\left(1+\alpha_{k,i}^{o}\right)p_{k,i}}h_{k,i}\chi_{k,i}^{o}-\left(D_{k}-\lambda_{k}I\right)w_{k}^{\left(t\right)}\right)$$

The iterative process continues until the difference between $w_{k}^{\left(t+1\right)}$ and $w_{k}^{\left(t\right)}$ falls below a predefined threshold, marking $w_{k}^{o}$ as the optimal solution to the unconstrained problem. Leveraging Proposition 2 from Ref. [15], the optimal solution for the original constrained problem is expressed as follows:

(22) $${\left(w_{k}^{o}\right)}^{\prime }=\sqrt{\frac{P_{max}}{\sum_{k=1}^{K}\sum_{i=1}^{2}p_{k,i}{\left\|w_{k}^{o}\right\|}^{2}}}w_{k}^{o},\forall k$$

The original optimization of $w_{k}$ involves matrix inversion, with a computational complexity of $O\left(K^{3}\right)$, which poses challenges for large-scale systems. In contrast, the proposed algorithm employs an iterative update rule, reducing the complexity to $O\left(T\cdot K^{2}\right)$, where T denotes the number of iterations, typically much smaller than K. This reduction in complexity improves the efficiency and scalability of the optimization. Algorithm 1 summarizes the proposed active beamforming optimization method.
**Algorithm 1:** Iterative Framework for Active Beamforming Optimization**Input:** channel vectors: $h_{k,i}$$\left(\forall k,i\right)$ Power allocation vectors: $p_{k,i}$$\left(\forall k,i\right)$; Noise variance: $\sigma^{2}$; Maximum iteration times: $T_{max}$; Convergence threshold: ε. **Output:** Optimal precoding vectors: $w_{k}$$\left(\forall k\right)$;
t=0.**While** $t<T_{max}$ doObtain the optimal $\left\{a_{k,i}^{\left(t\right)}\right\}$ according to (8);Obtain the optimal $\left\{\chi_{k,i}^{\left(t\right)}\right\}$ according to (11);Obtain the optimal $\left\{w_{k}^{\left(t\right)}\right\}$ according to (20);Check convergence: If $\left\|w_{k}^{\left(t+1\right)}+w_{k}^{\left(t\right)}\right\|\leq \varepsilon$, terminate the iteration process and consider $w_{k}^{\left(t+1\right)}$ as the optimal solution.$\mathrm{If}\;t\geq T_{\max}$$,\;\mathrm{stop}\;\mathrm{the}\;\mathrm{iteration}\;\mathrm{and}\;\mathrm{output}\;\mathrm{the}\;\mathrm{current}\;w_{k}^{\left(t+1\right)}$. Otherwise, set t=t+1 and go back to Step 3.**end while**return ${\left(w_{m}^{\circ }\right)}^{\prime }=\sqrt{\alpha }w_{m}^{\left(t+1\right)}$ for ∀m.

### 3.2. Passive Beamforming Optimization for STAR-RIS NOMA

Building upon the optimal precoding vectors derived in the previous section, this section we will focus on optimizing the passive beamforming for STAR-RIS NOMA systems. The proposed framework aims to optimize both the phase shifts and amplitude coefficients, as described below.

The passive beamforming optimization problem is formulated as follows:

(23a) $$\underset{\left\{v_{n}\right\}}{\max}\;f_{FP}\left(v\right)$$

(23b)  s.t. (6b), (6c), (6d)

where $f_{FP}(v)$ is expressed as follows:

(24) $$f_{FP}(v)=\overset{K}{\underset{k=1}{\sum }}\overset{2}{\underset{i=1}{\sum }}\left[2\sqrt{\left(1+\alpha_{k,i}^{o}\right)p_{k,i}}Re\left\{{\left(\chi_{k,i}^{o}\right)}^{H}h_{k,i}^{H}w_{k}\right\}-{\left|\chi_{k,i}^{o}\right|}^{2}\sum_{l=1}^{i}p_{k.l}{\left|h_{k,i}^{H}w_{k}\right|}^{2}-{\left|\chi_{k,i}^{o}\right|}^{2}\sigma_{k}^{2}\right]$$

For simplicity, define $q_{k,i}^{H}=diag(f_{k,i}^{H})g_{k}\in {\mathbb{C}}^{M\times L}$. Substituting this into Equation (24) and removing the terms unrelated to $\Theta^{n}={\left[\theta_{1}^{n},\theta_{2}^{n},\cdots ,\theta_{M}^{n}\right]}^{T}$ and $\beta^{n}={\left[\beta_{1}^{n},\beta_{2}^{n},\cdots ,\beta_{M}^{n}\right]}^{T}$, the expression can be simplified as follows:

(25) $$f\left(\theta ,\beta \right)=\overset{K}{\underset{k=1}{\sum }}\overset{2}{\underset{i=1}{\sum }}2Re\left\{E_{k,i}{\left(\Theta^{n}\right)}^{H}diag\left(\beta^{n}\right)q_{k,i}^{H}w_{k}\right\}-\overset{K}{\underset{k=1}{\sum }}\overset{2}{\underset{i=1}{\sum }}\left[F_{k,i}{\left\|{\left(\Theta^{n}\right)}^{H}\cdot diag\left(\beta^{n}\right)q_{k,i}^{H}w_{k}\right\|}^{2}\right]$$

where

(26a) $$E_{k,i}=\sqrt{\left(1+\alpha_{k,i}^{o}\right)p_{k,i}}\chi_{k,i}^{o}-{\left|\chi_{k,i}^{o}\right|}^{2}\sum_{l=1}^{i}p_{k,l}w_{k}^{H}h_{k,i}^{d}\in C^{1\times 1}$$

(26b) $$F_{k,i}={\left|\chi_{k,i}^{o}\right|}^{2}\sum_{l=1}^{i}p_{k,l}\in R^{1\times 1}$$

(26c) $$A_{k,i}=q_{k,i}^{H}w_{k}\in {\mathbb{C}}^{M\times 1}$$

and $A_{k,i}\left[m\right]$ denotes the m-th entry of the column vector $A_{k,i}$.

The coupling between the phase $\Theta^{n}$ and amplitude coefficients $\beta^{n}$ keeps the problem non-convex. To address this, we adopt an alternating optimization strategy, first fixing $\beta^{n}$ to optimize $\Theta^{n}$ and then fixing $\Theta^{n}$ to optimize $\beta^{n}$, simplifying the problem and ensuring convergence to a local optimal solution.

#### 3.2.1. Phase Optimization for Binary θ

Under the same power conditions, the system’s sum rate increases as the channel gain improves. Consequently, we can optimize the phase shifts of the STAR-RIS by maximizing the total equivalent channel gain. Furthermore, the transmission phase shift vector $\Theta^{t}$ and the reflection phase shift vector $\Theta^{r}$ are independent within their respective domains, meaning that they can be optimized separately. At this point, the optimization problem can be formulated as follows:

(27a) $$\underset{\left\{\theta_{m}^{n}\right\}}{\max}\overset{K}{\underset{k=1}{\sum }}\overset{2}{\underset{i=1}{\sum }}{\left\|h_{k,i}^{H}w_{k}\right\|}^{2}$$

(27b)  s.t. (6c), (6d)

Since the focus of this study is on binary phase shifts, we assume the transmission phase is restricted to $\varphi_{m}^{t}\in \left\{0^{\circ },180^{\circ }\right\}$, i.e., $\theta_{m}^{t}\in \left\{-1,1\right\},\forall m$. Under Constraint Equation (6d), the reflection phase is limited to $\varphi_{m}^{r}\in \left\{90^{\circ },270^{\circ }\right\}$, i.e., $\theta_{m}^{r}\in \left\{-j,j\right\},\forall m$. It is important to highlight that the proposed algorithm is equally applicable to the reverse configuration, where the reflection phase is set to $\varphi_{m}^{r}\in \left\{0^{\circ },180^{\circ }\right\}$ and the transmission phase to $\varphi_{m}^{t}\in \left\{90^{\circ },270^{\circ }\right\}$. Under binary phase conditions, the optimization of the transmission and reflection phases is mutually independent due to their distinct constraints. To further analyze the system, it is crucial to evaluate and express the rates for users located in the transmission and reflection regions separately. The following section provides a detailed explanation for each case, addressing the unique characteristics and requirements of these two regions.

To simplify, for each $k,i\in \left\{T\right\}$, we define the following composite vector:

(28) $$B_{k,i}^{t}=\left[\begin{array}{c} {\left(h_{k,i}^{d}\right)}^{H}w_{k}\\ diag\left(\beta^{t}\right)q_{k,i}w_{k} \end{array}\right],x^{t}={\left[\begin{array}{cc} 1 & \Theta^{t} \end{array}\right]}^{T}$$

Thus, the optimization problem for transmission-region users can be expressed as follows:

(29) $$\underset{\left\{x_{m}^{t}\right\}\in \left\{1,-1\right\}}{\max}{\sum_{ki\in \left\{T\right\}}\left\|{\left(B_{k,i}^{t}\right)}^{H}x^{t}\right\|}^{2}$$

where $x^{t}={\left[\begin{array}{cccc} 1, & x_{1}^{t}, & \cdots , & x_{M}^{t} \end{array}\right]}^{T}\in {\mathbb{C}}^{M+1}$.

For reflection-region users $k,i\in \left\{R\right\}$, the optimization problem for users located in the reflection region can be expressed as follows:

(30a) $$B_{k,i}^{r}=\left[\begin{array}{c} {\left(h_{k,i}^{d}\right)}^{H}w_{k}\\ diag\left(\beta^{r}\right)q_{k,i}w_{k} \end{array}\right],x^{r}={\left[\begin{array}{cc} 1 & \Theta^{r} \end{array}\right]}^{T}$$

(30b) $$\underset{\left\{x_{m}^{r}\right\}\in \left\{j,-j\right\}}{\max}{\sum_{ki\in \left\{R\right\}}\left\|{\left(B_{k,i}^{r}\right)}^{H}x^{r}\right\|}^{2}$$

where $\theta_{0}^{r}=x_{0}^{r}=1,\theta_{m}^{r}=x_{m}^{r},x^{r}={\left[1\begin{array}{cccc} , & x_{1}^{r}, & \cdots , & x_{M}^{r} \end{array}\right]}^{T},x_{m}^{r}\in \left\{j,-j\right\}$, since $x_{m}^{r}$ takes values from −j and j, to enable its optimization within the same range as $x_{m}^{t}$, the following transformation is applied: $x^{r}=j{\hat{x}}^{r},{\hat{x}}_{m}^{r}\in \left\{-1,1\right\}$. By substituting ${\hat{x}}^{r}$ into Equation (30b), the equation can be reformulated as follows:

(31) $$\underset{\left\{{\hat{x}}_{m}^{r}\right\}\in \left\{1,-1\right\}}{\max}{\sum_{ki\in \left\{R\right\}}\left\|{\left(-jB_{k,i}^{r}\right)}^{H}{\hat{x}}^{r}\right\|}^{2}$$

We rewrite each $B_{k,i}^{n}\left[m\right],m\in M,n\in \left\{t,r\right\}$ as an $R^{2}$ real-valued vector:

(32a) B^k,itm=ReBk,itmHImBk,itmH∈ℝ2,B^k,it=B^k,it0B^k,it1⋯B^k,itM

(32b) B^k,irm=Re−jBk,irmHIm−jBk,irmH∈ℝ2,B^k,ir=B^k,ir0B^k,ir1⋯B^k,irM

Leveraging the properties of norms, a dual variable y can be introduced for each vector ${\left({\hat{B}}_{k,i}^{t}\right)}^{H}x^{t}$ and ${\left({\hat{B}}_{k,i}^{r}\right)}^{H}{\hat{x}}^{r}$, respectively, ensuring that $\left\|y\right\|=1$ is satisfied. For each $k,i\in \left\{T\right\}$, leveraging the properties of norms, we can apply the Cauchy–Schwarz inequality to the norm of each term in the objective function:

(33) $${\sum_{ki\in \left\{T\right\}}\left\|{\left({\hat{B}}_{k,i}^{t}\right)}^{H}x^{t}\right\|}^{2}{\left\|y\right\|}^{2}\geq {\sum_{ki\in \left\{T\right\}}\left({\left({\left({\hat{B}}_{k,i}^{t}\right)}^{H}x^{t}\right)}^{T}y\right)}^{2}\geq {\left(\sum_{ki\in \left\{T\right\}}{\left({\left({\hat{B}}_{k,i}^{t}\right)}^{H}x^{t}\right)}^{T}y\right)}^{2}$$

Maximizing $x_{m}^{t}\in \left\{-1,1\right\}$ and $\left\|y\right\|=1$ gives

(34) $$\underset{x_{m}^{t}\in \left\{-1,1\right\},\left\|y\right\|=1}{\max}{\sum_{ki\in \left\{T\right\}}\left\|{\left({\hat{B}}_{k,i}^{t}\right)}^{H}x^{t}\right\|}^{2}{\left\|y\right\|}^{2}=\underset{x_{m}^{t}\in \left\{-1,1\right\},\left\|y\right\|=1}{\max}{\left(\sum_{ki\in \left\{T\right\}}{\left({\left({\hat{B}}_{k,i}^{t}\right)}^{H}x^{t}\right)}^{T}y\right)}^{2}$$

In this case, the equality in Equation (33) is achieved through the collaborative optimization of $x^{t}$ and y. Specifically, $x^{t}$ determines the discrete properties of the input signal and forms the foundational structure of the objective function, while y aligns with the direction of $\sum_{ki\in \left\{T\right\}}{\left({\hat{B}}_{k,i}^{t}\right)}^{H}x^{t}$ to ensure that the Cauchy–Schwarz inequality holds as an equality. This alignment enables the objective function to reach its theoretical maximum, demonstrating how $x^{t}$ and y play complementary roles in achieving optimization under the given constraints. The objective function can be formulated as

(35) $$\underset{x_{m}^{t}\in \left\{-1,1\right\},\left\|y\right\|=1}{\max}{\left(\sum_{ki\in \left\{T\right\}}{\left({\left({\hat{B}}_{k,i}^{t}\right)}^{H}\left[0\right]\right)}^{T}y+\sum_{ki\in \left\{T\right\}}x_{1}^{t}{\left({\left({\hat{B}}_{k,i}^{t}\right)}^{H}\left[1\right]\right)}^{T}y+\cdots +\sum_{ki\in \left\{T\right\}}x_{M}^{t}{\left({\left({\hat{B}}_{k,i}^{t}\right)}^{H}\left[M\right]\right)}^{T}y\right)}^{2}$$

The purpose of introducing y is to represent the complex objective function as a directional projection, thereby providing freedom of direction to achieve the maximum value of the objective.

The same principle can be used for reflection users as well. And the objective function can be expressed as follows:

(36) $$\underset{{\hat{x}}_{m}^{r}\in \left\{-1,1\right\},\left\|y\right\|=1}{\max}{\left(\sum_{ki\in \left\{R\right\}}\left(-j\right){\left({\left({\hat{B}}_{k,i}^{r}\right)}^{H}\left[0\right]\right)}^{T}y+\sum_{ki\in \left\{R\right\}}{\hat{x}}_{1}^{r}{\left({\left({\hat{B}}_{k,i}^{r}\right)}^{H}\left[1\right]\right)}^{T}y+\cdots +\sum_{ki\in \left\{R\right\}}{\hat{x}}_{M}^{r}{\left({\left({\hat{B}}_{k,i}^{r}\right)}^{H}\left[M\right]\right)}^{T}y\right)}^{2}$$

The problem is reformulated as a joint optimization over $\left(x,y\right)$. To address this, a decoupled approach is employed, wherein x and y are alternately optimized, while keeping the other variable fixed.

To simplify the expressions, we define the following:

(37a) $$v_{0}^{t}=\sum_{ki\in \left\{T\right\}}{\left({\hat{B}}_{k,i}^{t}\right)}^{H}\left[0\right],v_{1}^{t}=\sum_{ki\in \left\{T\right\}}{\left({\hat{B}}_{k,i}^{t}\right)}^{H}\left[1\right],\cdots ,v_{M}^{t}=\sum_{ki\in \left\{T\right\}}{\left({\hat{B}}_{k,i}^{t}\right)}^{H}\left[M\right]$$

(37b) $$v_{0}^{r}=-j\sum_{ki\in \left\{R\right\}}{\left({\hat{B}}_{k,i}^{r}\right)}^{H}\left[0\right],v_{1}^{r}=\sum_{ki\in \left\{R\right\}}{\left({\hat{B}}_{k,i}^{r}\right)}^{H}\left[1\right],\cdots ,v_{M}^{r}=\sum_{ki\in \left\{R\right\}}{\left({\hat{B}}_{k,i}^{r}\right)}^{H}\left[M\right]$$

Based on the above assumptions, the objective function is transformed into the following form.

(38a) $$\underset{x_{m}^{t}\in \left\{-1,1\right\},\left\|y\right\|=1}{\max}{\left({\left(v_{0}^{t}\right)}^{T}y+x_{1}^{t}{\left(v_{1}^{t}\right)}^{T}y+\cdots +x_{M}^{t}{\left(v_{M}^{t}\right)}^{T}y\right)}^{2}$$

(38b) $$\underset{{\hat{x}}_{m}^{r}\in \left\{-1,1\right\},\left\|y\right\|=1}{\max}{\left({\left(v_{0}^{r}\right)}^{T}y+{\hat{x}}_{1}^{r}{\left(v_{1}^{r}\right)}^{T}y+\cdots +{\hat{x}}_{M}^{r}{\left(v_{M}^{r}\right)}^{T}y\right)}^{2}$$

The subsequent section describes the optimization process for x. When y is fixed, the optimization of x for the binary phase shifts corresponds to determining its range for the associated components. Since the transmission and reflection optimizations are independent, they can be performed separately.

For transmission users, the optimization for transmission users is expressed as follows:

(39) $$f(x^{t})={\left(v_{0}^{t}\right)}^{T}{\left(y^{t}\right)}^{o}+x_{1}^{t}{\left(v_{1}^{t}\right)}^{T}{\left(y^{t}\right)}^{o}+\cdots +x_{M}^{t}{\left(v_{M}^{t}\right)}^{T}{\left(y^{t}\right)}^{o}$$

To ensure that x maximizes the objective function, it should be selected such that $v_{0}^{t}y^{o}$ and $v_{m}^{t}y^{o}$ are aligned in the same direction. Consequently, the optimal solution is as follows:

(40) $${\left(x_{m}^{t}\right)}^{o}=\mathrm{sgn}({\left(v_{0}^{t}\right)}^{T}{\left(y^{t}\right)}^{o}{\left(v_{m}^{t}\right)}^{T}{\left(y^{t}\right)}^{o})$$

which leads to the followsing optimal expression:

(41) $${\left(\theta_{m}^{t}\right)}^{o}=\arccos\left({\left(x_{m}^{t}\right)}^{o}\right)$$

For the reflection users, when y is fixed, the objective function becomes the following:

(42) $$f({\hat{x}}^{r})={\left(v_{0}^{r}\right)}^{T}{\left(y^{r}\right)}^{o}+{\hat{x}}_{1}^{r}{\left(v_{1}^{r}\right)}^{T}{\left(y^{r}\right)}^{o}+\cdots +{\hat{x}}_{M}^{r}{\left(v_{M}^{r}\right)}^{T}{\left(y^{r}\right)}^{o}$$

Similarly, the value of x should be selected to ensure that ${\left(v_{0}^{r}\right)}^{T}{\left(y^{r}\right)}^{o}$ and ${\left(v_{m}^{r}\right)}^{T}{\left(y^{r}\right)}^{o}$ are aligned, which will maximize the objective function. The optimal solution is as follows:

(43) $${\left({\hat{x}}_{m}^{r}\right)}^{o}=\mathrm{sgn}({\left(v_{0}^{r}\right)}^{T}{\left(y^{r}\right)}^{o}{\left(v_{m}^{r}\right)}^{T}{\left(y^{r}\right)}^{o})$$

with the optimal expression written as follows:

(44) $${\left({\hat{\theta }}_{m}^{r}\right)}^{o}=\arccos\left({\left({\hat{x}}_{m}^{r}\right)}^{o}\right)$$

Since $x_{m}^{r}=j{\hat{x}}_{m}^{r}$, it follows that:

(45) $${\left(\theta_{m}^{r}\right)}^{o}=\arccos\left(j{\left({\hat{x}}_{m}^{r}\right)}^{o}\right)$$

After obtaining the optimal solution for x, the next step is to optimize y.

For the case where $ki\in \left\{T\right\}$, assuming that $x^{t}$ is fixed, with each element constrained to predefined binary values (1 or −1), it now becomes an optimization problem concerning y. This reformulation simplifies the problem to identifying the optimal y that maximizes the objective function, given as follows:

(46) $$\underset{\left\|y\right\|=1}{\max}{\left(\left|{\left(v_{0}^{t}\right)}^{T}y\right|+\left|{\left(v_{1}^{t}\right)}^{T}y\right|+\cdots +\left|{\left(v_{M}^{t}\right)}^{T}y\right|\right)}^{2}$$

Similarly, for users on the reflective side, we can also draw the following conclusions.

(47) $$\underset{\left\|y\right\|=1}{\max}{\left(\left|{\left(v_{0}^{r}\right)}^{T}y\right|+\left|{\left(v_{1}^{r}\right)}^{T}y\right|+\cdots +\left|{\left(v_{M}^{r}\right)}^{T}y\right|\right)}^{2}$$

In this new problem, any y with a fixed magnitude is assumed to take either a positive or negative sign, resulting in $2^{M+1}$ possible sign combinations for the entire objective function. This still results in a high computational complexity. After determining the signs, the objective function can be efficiently maximized in linear time within the unit circle. Following the approach in Ref. [17], the unit circle can be partitioned for further analysis. Specifically, since y is a unit vector in a two-dimensional plane, its potential directions can be analyzed through segmentation.

**Figure 2:**
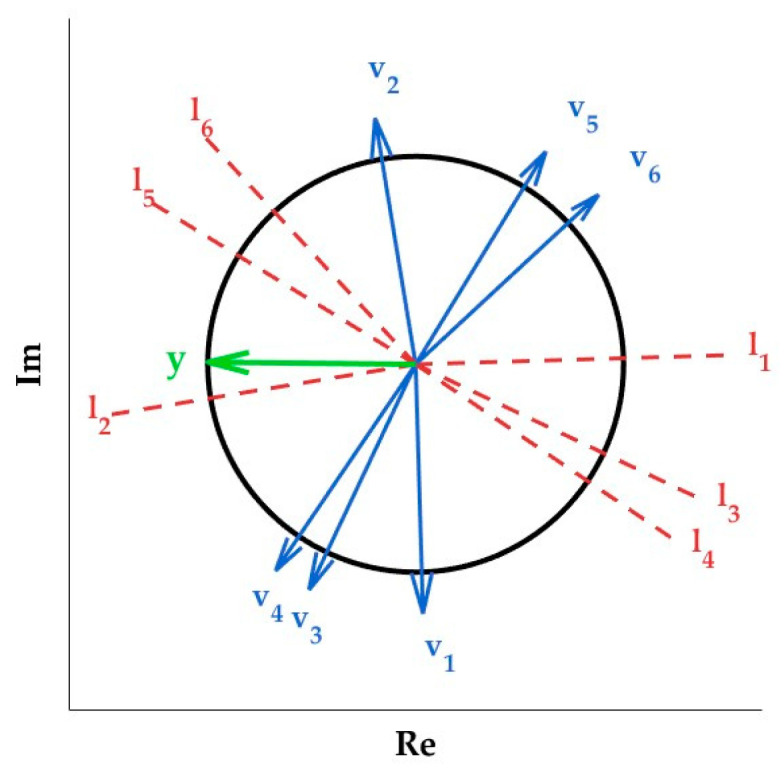
The binary phase beamforming.

As illustrated in Figure 2, let $v_{m}^{n},m=0,1,\cdots ,M,n\in \left\{t,r\right\}$ represent a set of normal vectors, and let their corresponding unique tangent lines be $l_{i},i=1,\cdots ,F$. Since multiple normal vectors can share the same tangent line, the total count of tangent lines F is constrained to F≤M+1. Let ${ϒ}_{i}^{n},n\in \left\{t,r\right\}$ represent the set of all normal line $v_{m}^{n}$, m=0,1,⋯,M corresponding to the $l_{i},i=1,\cdots ,F$. Among these, we let $l_{1}$ represent an arbitrary tangent line. Moving counterclockwise from $l_{1}$, label the remaining tangent lines sequentially as $l_{2},\cdots ,l_{F}$.These tangent lines partition the unit circle into 2F circular segments, denoted as $L_{1},L_{2},\cdots ,L_{2F}$. The segment $L_{1}$ is defined as the first segment intersected by both $l_{1}$ and $l_{2}$, while the remaining segments are labeled sequentially in the counterclockwise direction.

Each circular arc becomes a discrete region, reducing the search space of the optimization problem from the “entire unit circle” to the “finite 2F circular arcs”. Analyzing F arcs is sufficient, as the remaining F arcs are symmetric or identical. For each region $L_{l},l=1,\cdots ,F$, the optimal choice of ${\left(x_{m}^{t}\right)}^{o},{\left({\hat{x}}_{m}^{r}\right)}^{o}\in \left\{-1,1\right\}$ is fixed, and the objective function is a piecewise linear function of y. When y is confined to a specific segment $L_{l}$, simplifying the optimization problem can be achieved as follows:

(48) $$\underset{y\in {\mathbb{R}}^{2},\left\|y\right\|=1}{\max}{\left(B_{l}^{n}\right)}^{T}y$$

where

(49) $$B_{l}^{n}=\mathrm{sgn}\left(v_{0}^{t}{y_{l}}^{\prime }\right)v_{0}^{t}+\cdots +\mathrm{sgn}\left(v_{M}^{t}y_{l}\prime \right)v_{M}^{t}$$

where ${y_{l}}^{\prime }\in {\mathbb{R}}^{2}$ is any vector situated within the interior of $L_{l}$. We define ${\left(B_{l}^{n}\right)}^{T}y$ as the direction that maximizes the projection of $B_{l}^{n}$ along y, where this direction maximizes the projection of $B_{l}^{n}$. A straightforward approach is to compute each $B_{l}^{n}$ directly using Equation (43), which would require $O\left(M\right)$ time for each computation; this would result in $O\left(FM\right)$ time in total for all l=1,⋯,F. Our algorithm is capable of reducing the computational complexity to $O\left(M\right)$ by employing an iterative updating approach. Specifically, when the segment shifts from $L_{l}$ to $L_{l+1}$, it only requires updating the values of the ${\left(x_{m}^{n}\right)}^{o}$ variables related to the tangent line $l_{l+1}$, which separates the two segments, i.e., $\left\{{\left(x_{m}^{n}\right)}^{o}:\forall m\in {ϒ}_{l+1}^{n}\right\}$. Specifically, $v_{m}^{n}$ are affected, and the update rule is given by the following:

(50) $$B_{l+1}^{n}=B_{l}^{n}-\sum_{m\in {ϒ}_{l+1}^{n}}2\cdot \mathrm{sgn}\left(v_{m}^{n}y_{l}\prime \right)v_{m}^{n}$$

When $v_{m}^{n}$ lies in $L_{l}$, the corresponding sign is $\mathrm{sgn}\left(v_{m}^{n}y_{l}\prime \right)$. Therefore, when computing $L_{l+1}$, we just need to reverse the signs and subtract the original values, which is the same as subtracting twice.

The computation of the initial vector $B_{1}$ is $O\left(M\right)$. During subsequent iterations, we only need to compute points related to the new tangent $L_{l+1}$, resulting in a total complexity of $O\left(M\right)$.

Since our objective is $\underset{y\in {\mathbb{R}}^{2},\left\|y\right\|=1}{\max}{\left(B_{l}^{n}\right)}^{T}y$, we handle it as follows:

- Case 1: When y lies on the boundary of the unit circle and aligns (or opposes) with the direction of $B_{l}^{n}$, the objective function is maximized as ${\left(B_{l}^{n}\right)}^{T}y\leq \left\|B_{l}^{n}\right\|\cdot \left\|y\right\|$, which satisfies $\left\|y\right\|=1$. In this case, the optimal solution is as follows:

(51) $${\left(y_{l}^{n}\right)}^{o}=\frac{B_{l}^{n}}{\left\|B_{l}^{n}\right\|}$$

- Case 2: When the solution does not lie on the boundary of the unit circle, the optimization still occurs on the unit circle. However, the optimal direction deviates from $B_{l}^{n}$ and is the closest unit vector to $B_{l}^{n}$, given by the following:

(52) $${\left(y_{l}^{n}\right)}^{o}=\arg\underset{y\in {\mathbb{R}}^{2},\left\|y\right\|=1}{\min}\left\|y-\frac{B_{l}^{n}}{\left\|B_{l}^{n}\right\|}\right\|$$

By optimizing over each segment $L_{l},l=1,\cdots ,F$, we obtain the corresponding solutions $y_{1}^{0},\cdots ,y_{F}^{0}$. Since the objective function depends on y, this process ultimately yields the following global solution:

(53) $${\left(y^{n}\right)}^{o}=\max\left\{{\left(y_{1}^{n}\right)}^{o},\cdots ,{\left(y_{F}^{n}\right)}^{o}\right\}$$

Once phase optimization is complete, the next focus of optimization will be on coefficient optimization. This process will be outlined in the subsequent step.

#### 3.2.2. Amplitude Coefficient Optimization for β

Given that $\Theta^{n}$ is known, the objective function can be reformulated as follows:

(54a) $$\underset{\beta }{\min-}f\left(\beta \right)$$

(54b)  s.t. (6b).

where

(55) $$f_{FP}\left(\beta \right)=\overset{K}{\underset{k=1}{\sum }}\overset{2}{\underset{i=1}{\sum }}2Re\left\{E_{k,i}{\left(\Theta^{n}\right)}^{H}\cdot diag\left(\beta^{n}\right)A_{k.i}\right\}-\overset{K}{\underset{k=1}{\sum }}\overset{2}{\underset{i=1}{\sum }}\left[F_{k,i}{\left\|{\left(\Theta^{n}\right)}^{H}\cdot diag\left(\beta^{n}\right)A_{k,i}\right\|}^{2}\right]$$

To address the non-convexity of the problem caused by the constraints, inspired by Refs. [18,19], we introduce auxiliary variables. Consequently, the problem is equivalently reformulated as follows:

(56a) $$\underset{\beta^{t},\beta^{r},{\hat{\beta }}^{t},{\hat{\beta }}^{r}}{\min}-f\left(\beta \right)$$

(56b) $$s.t.{\hat{\beta }}_{m}^{t}=\beta_{m}^{t},{\hat{\beta }}_{m}^{r}=\beta_{m}^{r},m\in M$$

(56c) $${\left({\hat{\beta }}_{m}^{r}\right)}^{2}+{\left({\hat{\beta }}_{m}^{t}\right)}^{2}=1,m\in M$$

(56d) $$0\leq {\hat{\beta }}_{m}^{r}\leq 1,0\leq {\hat{\beta }}_{m}^{t}\leq 1,m\in M$$

In this reformulated problem, the constraints for $\beta^{t},\beta^{r}$ are linear, indicating that the optimization of $\beta^{t}$ and $\beta^{r}$ is independent. To incorporate the equality constraints into the objective function, the problem is reformulated as an augmented Lagrangian (AL) problem:

(57) $$\underset{\beta^{t},\beta^{r},{\hat{\beta }}^{t},{\hat{\beta }}^{r}}{\min}-f\left(\beta \right)+\frac{1}{2\varsigma }\sum_{m=1}^{M}\left({\left\|{\hat{\beta }}_{m}^{t}-\beta_{m}^{t}+\varsigma \lambda_{m}^{t}\right\|}^{2}+{\left\|{\hat{\beta }}_{m}^{r}-\beta_{m}^{r}+\varsigma \lambda_{m}^{r}\right\|}^{2}\right)$$

where ς>0 is the penalty factor, and $\lambda_{m}^{n},n\in \left\{t,r\right\}$ represents the Lagrangian dual variable. The optimization problem can be solved by alternately optimizing $\left\{\beta^{t},\beta^{r}\right\}$ and $\left\{{\hat{\beta }}^{t},{\hat{\beta }}^{r}\right\}$. The inclusion of penalty terms does not affect the convexity of the objective function. Consequently, the unconstrained subproblem for $\left\{\beta^{t},\beta^{r}\right\}$ is convex, expressed as follows:

(58) $$\underset{\beta^{t},\beta^{r}}{\min}-f_{FP}\left(\beta \right)+\frac{1}{2\varsigma }\left(\sum_{m=1}^{M}{\left\|{\hat{\beta }}_{m}^{t}-\beta_{m}^{t}+\varsigma \lambda_{m}^{t}\right\|}^{2}+{\left\|{\hat{\beta }}_{m}^{r}-\beta_{m}^{r}+\varsigma \lambda_{m}^{r}\right\|}^{2}\right)$$

As there is no coupling between $\beta^{t}$ and $\beta^{r}$ at this stage, their optimization is completely independent. Thus, the optimization problem is decomposed into two separate subproblems, and can each be solved independently by computing the derivatives. Specifically, the closed-form expressions for the optimal expressions are presented as follows:

(59a) βmt=2∑ki∈TReEk,iθmt*Ak,im−2Re∑ki∈TFk,i(θmtAk,i*m)H∑q≠mMβqtθqtAk,i*q+1ς(β^mt+ςλmt)2Re∑ki∈TFk,i(θmtAk,i*m)HθmtAk,i*m+1ς

(59b) βmr=2∑ki∈RReEk,iθmt*Ak,im−2Re∑ki∈RFk,i(θmrAk,i*m)H∑q≠mMβqrθqrAk,i*q+1ς(β^mr+ςλmr)2Re∑ki∈RFk,i(θmrAk,i*m)HθmrAk,i*m+1ς

Since $\left\{{\hat{\beta }}^{t},{\hat{\beta }}^{r}\right\}$ only appear in the penalty terms, the corresponding optimization problem can be reformulated as follows:

(60a) $$\underset{{\hat{\beta }}^{t},{\hat{\beta }}^{r}}{\min}\frac{1}{2\varsigma }\sum_{m=1}^{M}\left({\left\|{\hat{\beta }}_{m}^{t}-\beta_{m}^{t}+\frac{1}{2\varsigma }\lambda_{m}^{t}\right\|}^{2}+{\left\|{\hat{\beta }}_{m}^{r}-\beta_{m}^{r}+\frac{1}{2\varsigma }\lambda_{m}^{r}\right\|}^{2}\right)$$

(60b)  s.t. (57c),(57d)

Constraint Equation (57c) requires ${\hat{\beta }}_{m}^{n}$ to lie on the unit circle, while Constraint Equation (57d) further restricts it to the arc in the first quadrant. This reformulates the problem as projecting a vector onto the first-quadrant arc of the unit circle, resulting in an equivalent optimization problem. Specifically, we define a projection without an offset as follows:

(61) $$\varpi_{m}^{t}=\beta_{m}^{t}-\frac{1}{2\varsigma }\lambda_{m}^{t},\varpi_{m}^{r}=\beta_{m}^{r}-\frac{1}{2\varsigma }\lambda_{m}^{r}$$

The objective function becomes the following:

(62) $$\underset{{\hat{\beta }}^{t},{\hat{\beta }}^{r}}{\min}\sum_{m=1}^{M}\left({\left\|{\hat{\beta }}_{m}^{t}-\varpi_{m}^{t}\right\|}^{2}+{\left\|{\hat{\beta }}_{m}^{r}-\varpi_{m}^{r}\right\|}^{2}\right)$$

For each m, the optimization problem is independent, allowing us to solve it point by point. By projecting each point onto the unit circle arc in the first quadrant, the specific formulas are as follows:

(63) $${\hat{\beta }}_{m}^{t}=\cos({\hat{\theta }}_{m}),{\hat{\beta }}_{m}^{r}=\sin({\hat{\theta }}_{m}),where\;{\hat{\theta }}_{m}=\mathrm{arc}\tan2\left(\varpi_{m}^{r},\varpi_{m}^{t}\right)$$

Important notes: If ${\hat{\theta }}_{m}>\frac{\pi }{2}$, it must be adjusted as ${\hat{\theta }}_{m}=\min\left\{{\hat{\theta }}_{m},\frac{\pi }{2}\right\}$ to ensure that Constraint Equation (64) is satisfied.

The above transformation effectively converts the original problem into multiple independent subproblems, addressing the challenges posed by non-convexity, while ensuring constraint satisfaction. The optimization process for the transmission and reflection coefficient vectors is detailed in Algorithm 2.

**Algorithm 2:** Proposed Binary Phase Passive Beamforming Optimization Algorithm**Input:** Channel vectors: $h_{k,i}$$\left(\forall k,i\right)$; power allocation vectors: $p_{k,i}$$\left(\forall k,i\right)$; precoding vectors: $w_{k}$$\left(\forall k\right)$; noise variance: $\sigma^{2}$; maximum iteration times: $T_{max}$; convergence threshold:ε. **Output:** Optimal phase vectors: $\Theta^{n}\left(\forall n\right)$; optimal coefficient: $\beta_{m}^{n}\left(\forall m,n\right)$.
**Initialize**l $\Theta^{n}$$,\;\beta_{m}^{n}$.Compute the optimal $\Theta^{t}$ according to (50);Compute the optimal $\Theta^{r}$ according to (54);$\mathrm{Compute}\;\mathrm{the}\;\mathrm{optimal}\;\beta_{m}^{t}$ according to (60a);$\mathrm{Compute}\;\mathrm{the}\;\mathrm{optimal}\;\beta_{m}^{r}$ according to (60b);Check convergence: If $\left\|f_{FP}^{\left(t+1\right)}-f_{FP}^{\left(t\right)}\right\|\leq \varepsilon$,$\;\mathrm{terminate}\;\mathrm{the}\;\mathrm{iteration}\;\mathrm{process}\;\mathrm{and}\;\mathrm{consider}\;\left(\Theta ,\beta \right)$ as the optimal solution.$\mathrm{If}\;t\geq T_{\max}$$,\;\mathrm{stop}\;\mathrm{the}\;\mathrm{iteration}\;\mathrm{and}\;\mathrm{output}\;\mathrm{the}\;\mathrm{current}\;\left(\Theta ,\beta \right)$.$\mathrm{Otherwise},\;\mathrm{update}\;{\left(\Theta^{n}\right)}^{\left(t\right)}={\left(\Theta^{n}\right)}^{\left(t-1\right)}$$,\;{\left(\beta_{m}^{n}\right)}^{\left(t\right)}={\left(\beta_{m}^{n}\right)}^{\left(t-1\right)}$ and go back to Step 2.**end while**return $\left(\Theta ,\beta \right)$.

The detailed steps of the joint optimization process are presented in Algorithm 3.

**Algorithm 3:** Joint Active and Passive Beamforming Optimization**Input:** Channel vectors: $h_{k,i}$$\left(\forall k,i\right)$; power allocation vectors: $p_{k,i}$$\left(\forall k,i\right)$; precoding vectors: $w_{k}$$\left(\forall k\right)$; noise variance: $\sigma^{2}$; maximum iteration times: $T_{max}$; convergence threshold: ε. **Output:** Optimal active beamforming matrix: W; $\mathrm{Optimal}\;\mathrm{passive}\;\mathrm{beamforming}\;\mathrm{vector}:\;v_{n}$.
**Initialize**l $w_{k}$$,\;\Theta^{n}$$,\;\beta^{n}$.Repeat until convergence or $t\geq T_{max}$;**Optimize Active Beamforming:** $\mathrm{Fix}\;{\left(v_{n}\right)}^{\left(t\right)}$$\;\mathrm{and}\;\mathrm{compute}\;W^{\left(t+1\right)}$ using Algorithm 1;**Optimize Passive Beamforming:**$\;\mathrm{Fix}\;W^{\left(t+1\right)}$$\;\mathrm{and}\;\mathrm{compute}\;{\left(v_{n}\right)}^{\left(t+1\right)}$ using Algorithm 2;Check Convergence:$\mathrm{Compute}\;\mathrm{the}\;\mathrm{system}\;\mathrm{sum}\;\mathrm{rate}\;{R_{sum}}^{\left(t+1\right)}$;If $\left\|{R_{sum}}^{\left(t+1\right)}-{R_{sum}}^{\left(t\right)}\right\|\leq \varepsilon$,$\;\mathrm{terminate}\;\mathrm{the}\;\mathrm{iteration}\;\mathrm{process}\;\mathrm{and}\;\mathrm{output}\;W^{o}=W^{\left(t+1\right)},{\left(v_{n}\right)}^{o}={\left(v_{n}\right)}^{\left(t+1\right)}$.Otherwise, set t=t+1 and continue.**end while**return $W^{o},{\left(v_{n}\right)}^{o}$.

## 4. Numerical Results

This section presents the numerical results obtained from the simulations conducted on the proposed STAR-RIS-assisted NOMA system. These results reflect the system’s performance under various realistic conditions.

### 4.1. Simulation Setting

The simulation settings for evaluating the performance of the proposed STAR-RIS-assisted NOMA system are described as follows.

The simulation is conducted within a three-dimensional (3D) coordinate framework. The AP is positioned at the origin (0, 0, 0), while the STAR-RIS is strategically located at (50, 0, 20). Users are uniformly and randomly distributed within a circular area with a radius of 10 m, centered around the STAR-RIS.

The system operates at a frequency of 2.4 GHz with a total bandwidth of 1 MHz. The noise power is set to −80 dBm. The maximum transmit power is constrained to 2 W to optimize energy usage while maintaining system performance. The maximum transmit power is set as $P_{\max}=2\;W$; constrained to balance energy efficiency and performance, the optimization algorithms employ a convergence tolerance of $10^{-4}$ [6], and the maximum number of iterations is $T_{max}=500$. The simulations focus on precoding and phase shift optimization, independent of specific modulation schemes. The proposed framework is applicable to various modulation formats.

The communication channels are characterized by Rician fading, with a Rician factor of κ=3 dB, incorporating both line-of-sight (LoS) and non-line-of-sight (NLoS) components. To ensure statistical diversity in LoS and NLoS conditions, randomized user positions are considered. This choice is particularly relevant in STAR-RIS systems, where the LoS component significantly influences performance optimization. By adopting this approach, we validate the robustness of the proposed algorithm across practical scenarios, without being restricted to a specific propagation model. The path loss is modeled as

(64) $$L\left(d\right)=\rho_{0}{\left(\frac{d}{d_{0}}\right)}^{-\alpha }$$

where d is the distance between the transmitter and receiver; $d_{0}=1$ m (meter) is the reference distance; and $\rho_{0}$ represents the path loss at the reference distance, as follows:

(65) $$\rho_{0}={\left(\frac{\lambda }{4\pi d_{0}}\right)}^{2}={\left(\frac{0.125}{4\pi \cdot 1}\right)}^{2}\approx 0.001\left(-30\;dB\right)$$

α is the path loss exponent, set to $\alpha_{AP-RIS}=2.2$ for the AP-to-RIS link; $\alpha_{RIS-User}=2.8$ for the RIS-to-user links. The Rician fading sub-channels for the AP-to-STAR-RIS and STAR-RIS-to-user links are represented as follows. For the AP-to-STAR-RIS link, the channel is given by the following:

(66) $$g_{k}=\sqrt{L\left(d_{AS}\right)}\left(\sqrt{\frac{\kappa_{AS}}{\kappa_{AS}+1}}g_{k}^{LoS}+\sqrt{\frac{1}{\kappa_{AS}+1}}g_{k}^{NLoS}\right)$$

and for the STAR-RIS-to-user link, it is found by

(67) $$f_{k,i}=\sqrt{L\left(d_{SU,i}\right)}\left(\sqrt{\frac{\kappa_{SU}}{\kappa_{SU}+1}}f_{k,i}^{LoS}+\sqrt{\frac{1}{\kappa_{SU}+1}}f_{k,i}^{NLoS}\right)$$

Here, $d_{AS}$ is the distance between the AP and the RIS, while $d_{SU,i}$ represents the distance between the RIS and user i. Both links share a Rician factor of $\kappa_{AS}=\kappa_{SU}=3\;dB$. The LoS components $g_{k}^{LoS}$ and $f_{k,i}^{LoS}$ are deterministic, while the NLoS components $g_{k}^{NLoS}$ and $f_{k,i}^{NLoS}$ follow a Rayleigh fading distribution. This detailed modeling ensures that the channel dynamics and system configurations closely mirror practical scenarios, providing a realistic evaluation of the proposed STAR-RIS-assisted system.

To demonstrate the performance of the proposed schemes, we compare them with a baseline system:

Baseline 1 (STAR-RIS NOMA With 1-bit): For W, this system uses zero-forcing (ZF), while coherent beamforming adopts the nearest point projection (NPP) method from Ref. [20].

Baseline 2 (STAR-RIS NOMA With Continuous Phase): For W, this system uses ZF, while $v_{n}$ optimization assumes an ideal state, solved using the convex upper bound (CUB) method, allowing more freedom than that afforded by discrete 1-bit phase shifters.

### 4.2. Simulation Results

#### 4.2.1. Comparison of System Sum Rate at Different Iteration Numbers

**Figure 3:**
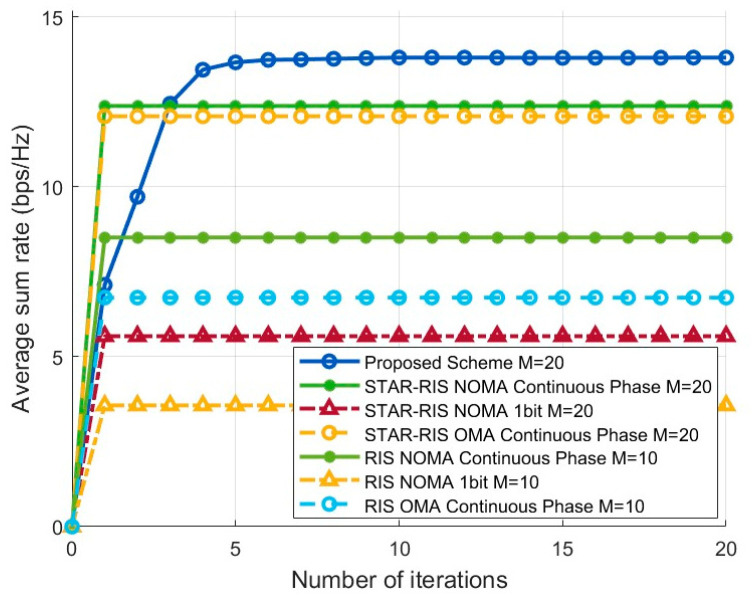
Sum-rate performance over iterations for K=6,M=20.

Figure 3 illustrates the variation in average sum rate with the number of iterations for K=6 and M=20. It is clear from the figure that the proposed scheme converges quickly, and it achieves the highest average sum rate throughout, significantly outperforming the other schemes. This demonstrates that the proposed scheme effectively enhances the optimization of both active and passive beamforming, resulting in improved system throughput. The STAR-RIS NOMA continuous phase and STAR-RIS OMA continuous phase optimization schemes converge the fastest but perform slightly worse than the proposed scheme. In contrast, the RIS schemes exhibit notably lower average sum rates, which are also significantly lower than their corresponding STAR-RIS counterparts. This is primarily due to the inherent limitations of RISs in terms of operational principles, coverage, and other factors. On the other hand, STAR-RISs provide significant performance improvements through advantages such as simultaneous transmission and reflection of signals, 360-degree coverage, enhanced signal strength, and flexible configuration protocols. These factors contribute to the substantial enhancement of system performance.

#### 4.2.2. Comparison of System Sum Rate at Different Element Numbers

**Figure 4:**
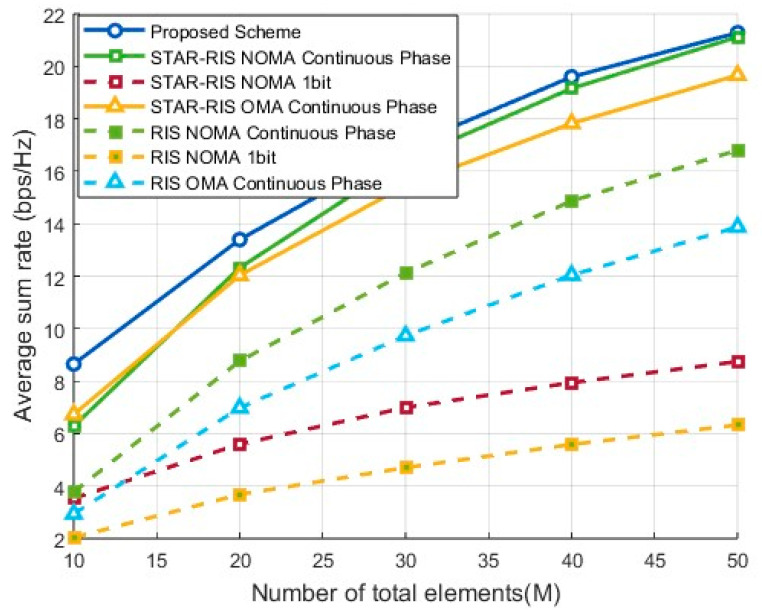
Sum-rate performance by number of elements.

Figure 4 illustrates the variation in system sum rate with the increase in the total number of elements M under the condition of six users for different optimization schemes. As M increases, the system sum rate of all schemes improves significantly due to the higher beamforming gain provided by more elements, which enhances both signal quality and coverage.

In the small-scale element scenario where M≤50, the proposed 1-bit discrete phase design demonstrates higher efficiency and performance, as it adopts a linear-time closed-form solution for the joint optimization of active and passive beamforming. This performance surpasses that of continuous phase optimization relying on the approximate solution of the CUB algorithm. As M increases, the continuous phase design gradually demonstrates its advantage via higher beamforming degrees of freedom, allowing for more precise phase control to further enhance signal gain. The performance gap with the proposed algorithm narrows, and it can be anticipated that the continuous phase design will eventually surpass the discrete design in large-scale scenarios. Additionally, STAR-RISs simultaneously optimize transmissive and reflective beamforming, effectively enhancing the flexibility and controllability of signal propagation. Compared to traditional RISs, STAR-RISs offer higher degrees of freedom, demonstrating significant advantages in controlling signal propagation directions and improving channel differentiation.

#### 4.2.3. Comparison of System Sum Rate at Different User Numbers

**Figure 5:**
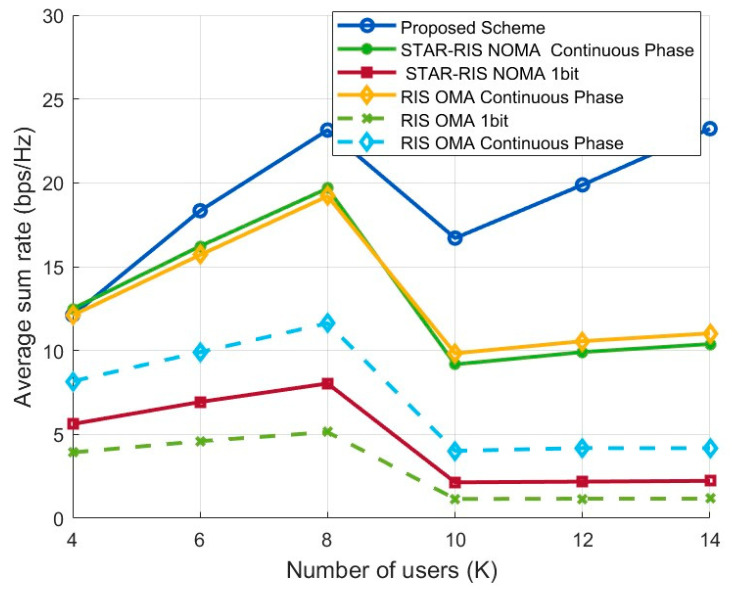
Sum-rate performance according to user numbers.

Figure 5 presents a comparison of the average sum-rate performance of the proposed STAR-RIS NOMA scheme and the baseline schemes under varying numbers of users (K). The simulation results show that the proposed scheme outperforms the baseline schemes, demonstrating its advantage in terms of spectral efficiency.

From the figure, it is easy to see that the sum rate of the proposed scheme, as well as the STAR-RIS NOMA continuous phase optimization scheme and the RIS OMA continuous phase optimization scheme, follows the trend of “initial increase, followed by a decrease, and then another increase.” However, the increase after the decline is more gradual for the latter two schemes compared to that of the proposed scheme. Specifically, when the number of users is small, the cooperation between NOMA power domain multiplexing and STAR-RIS dynamic phase optimization significantly enhances spectral efficiency. As the number of users increases, user interference intensifies, leading to a brief drop in the sum rate. At higher user numbers, the system reallocates resources through precoding design and phase vector optimization, and the multi-user diversity gain gradually emerges, resulting in the sum rate recovering and rising. Furthermore, for the STAR-RIS NOMA 1-bit phase quantization, limited phase resolution leads to significantly lower performance compared to that of the continuous phase optimization scheme and the proposed 1-bit optimization scheme, underscoring the necessity of precoding and phase optimization to improve the sum rate.

Although the simulation results indicate that our proposed optimization scheme outperforms the baseline schemes in terms of sum-rate performance, the performance fluctuations at high user numbers also highlight the challenges of interference management and resource allocation.

#### 4.2.4. Comparison of Transmission and Reflection Coefficients at Different Element Indices

**Figure 6:**
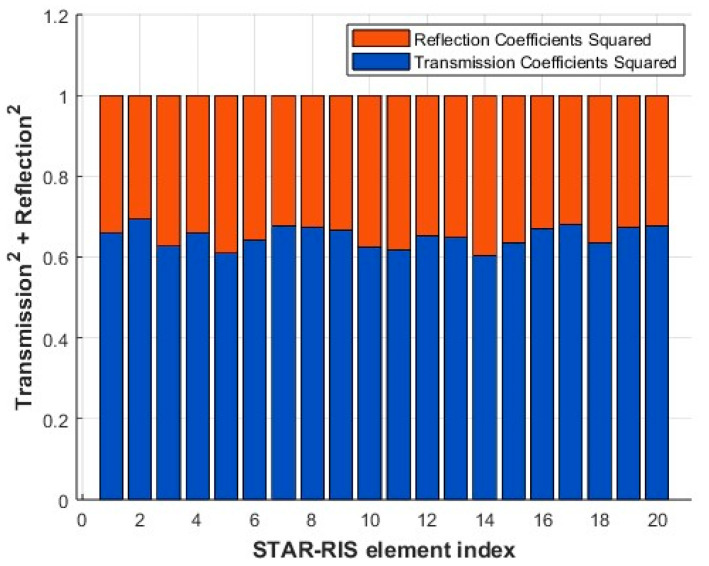
Amplitude control of transmitted and reflected signals.

From Figure 6, the transmission side of the STAR-RIS is allocated larger coefficients, as this region contains users with stronger channel gains in the given scenario. Traditional RISs struggle in NOMA networks when served users have similar channel conditions, limiting NOMA’s multiplexing gain. STAR-RISs overcome this limitation by dynamically adjusting the transmission and reflection coefficients for each element to optimize energy distribution. As shown in Figure 6, STAR-RISs concentrate more power on the transmission side, where users with higher decoding orders and stronger channel gains are located. This enhances NOMA’s multiplexing gain, boosts network throughput, and improves user differentiation in the transmission region.

In the illustrated case, six users are symmetrically distributed across the transmissive and reflective regions. After user pairing, STAR-RISs intelligently allocate greater energy to the transmission side, leveraging their superior channel conditions. This strategy ensures better energy efficiency and a high-quality communication experience for users.

#### 4.2.5. Performance Comparison of STAR-RIS NOMA Schemes in Terms of Average Sum Rate, Element Count, and Runtime

**Figure 7:**
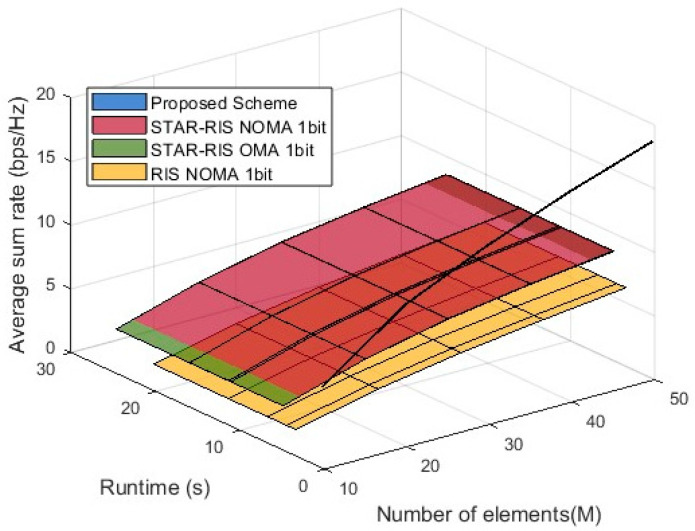
Performance comparison: average sum rate vs. element counts and runtime.

In Figure 7, the 3D simulation graph presents a performance comparison of different schemes in terms of average sum rate as a function of the number of STAR-RIS elements (M) and runtime. The proposed algorithm is marked in blue, and due to the short runtime required for optimization, the x-axis appears narrow, ultimately making the entire algorithm appear as a black line. It is evident from the graph that the proposed scheme consistently achieves a higher average sum rate than all of the baseline schemes, demonstrating its effectiveness in optimizing precoding and phase control, thereby maintaining a high sum rate across different runtime durations and values of M. Additionally, the surfaces representing STAR-RIS NOMA 1-bit and STAR-RIS OMA 1-bit are overall higher than that of RIS NOMA 1-bit, indicating the advantage of STAR-RIS technology in improving the sum rate. However, the impact of 1-bit quantization limits the performance of these two schemes, making them inferior to the proposed scheme. Furthermore, the results show that increasing the number of STAR-RIS elements enhances the sum rate for all schemes, but the proposed scheme exhibits a significantly greater improvement, fully validating its superiority in maximizing spectral efficiency in STAR-RIS NOMA systems.

#### 4.2.6. Performance Comparison of STAR-RIS NOMA Schemes in Terms of Average Sum Rate, Number of Users, and Runtime

**Figure 8:**
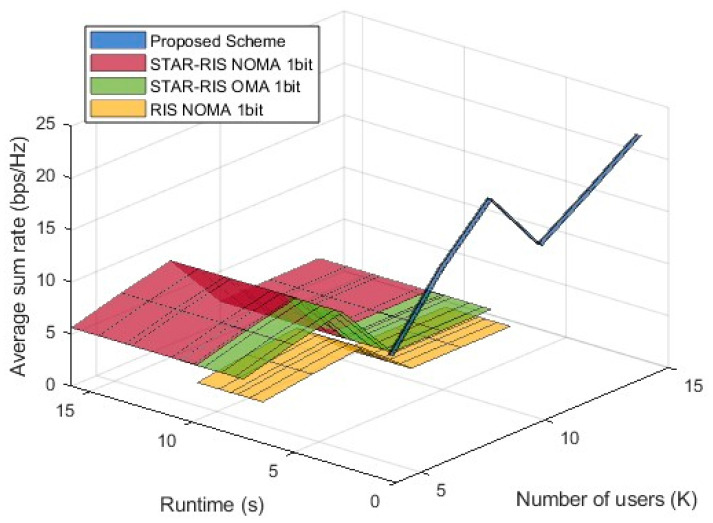
Performance comparison: average sum rate vs. number of users and runtime.

Figure 8 illustrates the trends of different schemes in terms of average sum rate as the number of users (K) and runtime varies. The proposed algorithm is marked in blue, and similarly, due to its short optimization runtime, the x-axis range appears narrow and concentrated near zero, resulting in a black jagged line. It is evident that the proposed scheme consistently achieves a higher average sum rate than all of the baseline schemes, confirming its effectiveness in optimizing precoding and phase control. STAR-RIS NOMA 1-bit and STAR-RIS OMA 1-bit are generally positioned above RIS NOMA 1-bit, indicating that STAR-RIS technology offers a significant advantage over conventional RIS in enhancing the sum rate. The steep rise in the proposed scheme’s curve suggests that as the number of users increases, the optimization strategy efficiently leverages the characteristics of STAR-RISs to achieve higher spectral efficiency. In contrast, the baseline schemes exhibit relatively stable performance due to the limitations imposed by 1-bit quantization. The overall trend shows that an increasing number of users contributes to sum rate improvement in all schemes, but the gain achieved by the proposed scheme is significantly higher than that of the baseline systems, demonstrating its superior adaptability and scalability in STAR-RIS NOMA systems. Figure 8 shows that the proposed scheme has a significant advantage over the baseline schemes in both runtime efficiency and sum rate enhancement.

## 5. Conclusions

This study investigates the joint optimization of active beamforming and passive phase modulation in STAR-RIS-assisted NOMA systems, aiming to maximize system throughput, while minimizing computational complexity. We develop an iterative optimization framework that decouples the original non-convex problem into two alternating subproblems: First, FP transforms active beamforming into an equivalent convex optimization problem, followed by the use of Nesterov’s extrapolation to reduce computational complexity. For the phase constraints, we propose a binary optimization strategy with unit modulus constraints, achieving closed-form solutions through geometric projection on the unit circle in the complex plane, with linear time complexity. The simulation results demonstrate that the proposed algorithm significantly outperforms the baseline methods for both performance and efficiency.

In future work, we will develop hybrid frameworks that combine machine learning with analytical optimization to improve the performance and interpretability of STAR-RIS-NOMA systems. While machine learning provides useful tools for handling dynamic environments, the explicit analytical relationships established in this study serve as a clear foundation for such integration. Additionally, we will refine K−ary phase control strategies to enhance system performance, while maintaining computational efficiency, allowing for more accurate beamforming and resource allocation.

## Data Availability

The data presented in this study are available on request from the corresponding author.
